# HIV-1 Tat and AIDS-associated cancer: targeting the cellular anti-cancer barrier?

**DOI:** 10.1186/1756-9966-27-3

**Published:** 2008-05-15

**Authors:** Giuseppe Nunnari, Johanna A Smith, René Daniel

**Affiliations:** 1 University of Catania, Department of Medicine and Medical Specialties, Division of Infectious Diseases, Via Palermo 636, Catania, Italy; 2 Center for Human Virology, Division of Infectious Diseases and Environmental Medicine, Department of Medicine, Thomas Jefferson University, 1020 Locust Street, Philadelphia, PA 19107, USA

## Abstract

The acquired immunodeficiency syndrome (AIDS) is accompanied by a significant increase in the incidence of neoplasms. Several causative agents have been proposed for this phenomenon. These include immunodeficiency and oncogenic DNA viruses and the HIV-1 protein Tat. Cancer in general is closely linked to genomic instability and DNA repair mechanisms. The latter maintains genomic stability and serves as a cellular anti-cancer barrier. Defects in DNA repair pathway are associated with carcinogenesis.

This review focuses on newly discovered connections of the HIV-1 protein Tat, as well as cellular co-factors of Tat, to double-strand break DNA repair. We propose that the Tat-induced DNA repair deficiencies may play a significant role in the development of AIDS-associated cancer.

## AIDS and cancer

The development of neoplasms in AIDS is generally assumed to be due to a failure of immune surveillance and infections by oncogenic viruses. However, a growing body of evidence suggests that HIV-1 and its proteins may play a more direct role in tumor development.

Several types of cancer have been strongly associated with the HIV-1 infection and AIDS. These are Kaposi sarcoma (KS), lymphoproliferative disorders and cervical cancer [1]. Kaposi sarcoma is the most common malignancy associated with HIV-1 infection [1]. It can be observed at any stage of the infection, although it occurs more frequently in later stages when severe immune suppression occurs [2]. KS is characterized by proliferation of spindle cells, which seem to be of lymphatic endothelial origin. A viral pathogenesis strongly contributes to KS and the human herpesvirus 8 (HHV-8 or KSHV – Kaposi's sarcoma-associated herpesvirus) DNA virus was found to be associated with KS [3-7]. In addition to HHV-8, it has been suggested that the HIV-1 protein Tat may contribute to the incidence and to the aggressive growth of KS [8]. Tat is released from HIV-1-infected cells and may bind to cell surfaces and enter uninfected cells [9-11]. One type of cells that Tat is known to target is endothelial cells. Tat induces angiogenesis and stimulates the growth of KS spindle cells [1,10,12-14]. Overexpression of Tat *in vivo *has been associated with the increase of KS-like lesions, although this role of Tat in KS development has been controversial [15]. In summary, Tat may act as a cofactor in KS development, although its role in KS is at present unclear.

Two other types of cancer have been associated with AIDS. HIV-1 infected patients develop lymphomas at relatively high frequency and in 3% of HIV-1 infected patients, non-Hodgkin lymphoma (NHL) is the initial manifestation of AIDS. As is the case of KS, NHL in HIV-1-infected patients was associated with a viral infection, in this case, Epstein-Barr virus infection (EBV) [12]. Finally, invasive cervical cancer is one of the most frequent complications of HIV-1 infection in developing countries [16]. It appears that cervical cancer in HIV-1 infected individuals is associated with the HPV infection, as is true for cervical cancer in general. Interestingly, the occurrence of this type of cancer is not dependent on immune suppression. The disease is aggressive in HIV-1 infected women, less responsive to treatment and often recurrent [16]. There is no difference in severity of cervical cancer in asymptomatic patients with HIV-1 infection and those with AIDS [16]. These data indicate that a failure of immune surveillance is not the only mechanism behind cancer development in AIDS, and that it may not be dependent on immune suppression. In contrast, it seems likely that HIV-1 infection and HIV-1 proteins contribute to cancer development in HIV-1-infected patients. This hypothesis is also supported by the suggested role of Tat in development of Kaposi sarcoma (see above). Although there is no evidence so far regarding the role of Tat in AIDS-associated NHL and cervical cancer, it cannot be excluded at present.

Finally, there are reports of increase in incidence of colorectal cancers in long-term AIDS survivors [17-22]. Colorectal cancer does not have a known viral etiology. Interestingly, Tat may play a role in development of this type of cancer in HIV-1-infected patients [23]. The mechanism underlying this effect of Tat has to be yet fully elucidated and we present below one possible mechanism how Tat may affect cellular processes, which are closely related to cancer development.

## Sensing and repair of double-strand DNA breaks

Cells have developed many mechanisms to protect the integrity of chromosomal DNA. These mechanisms are important for cell and organism survival. Surveillance protein complexes monitor the integrity of the genome. Detection of aberrant DNA and chromosome structures such as double-strand DNA breaks coordinately triggers checkpoint pathways and DNA repair systems [24]. Activation of a DNA damage checkpoint results in cell cycle arrest, allowing time for DNA repair. Cells posses many DNA repair systems, which are designed to recognize and repair specific types of DNA damage. For example, ionizing radiation induces single-strand and double-strand DNA breaks [25]. If such DNA damage is not repaired, it may result in the activation of programmed cell death (apoptosis) or mutations, such as oncogenic translocations.

The most hazardous form of DNA damage is a double-strand DNA break (DSB) [25-27]. Even one unrepaired DSB has been reported to kill a DNA repair-deficient cell [25-27]. In order to combat this potentially lethal DNA lesion, cells have developed several mechanisms to repair DSBs, which are important for cell and organism survival.

**Figure 1:**
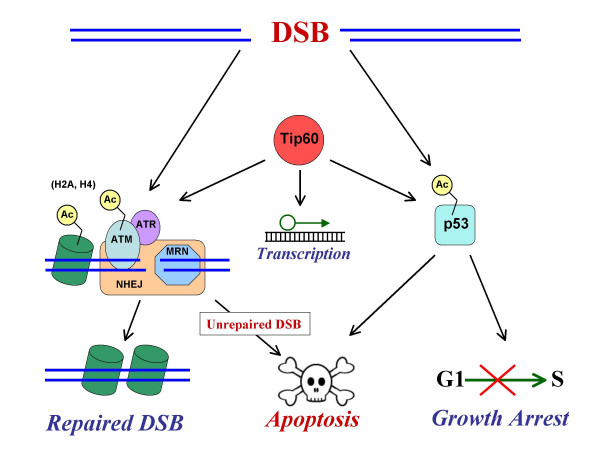
**Model for the role of Tip60 in DSB repair**. Tip60 appears to play a role in DSB repair through its acetyltransferase activity. Tip60 displays HAT (histone acetyltransferase) activity by acetylating histones H2A and H4 (nucleosomes are represented by green barrels) which may have important consequences in response to DSBs. Tip60 has been suggested to play a direct role in the activation ATM (a crucial DSB repair protein kinase) by acetylating it in response to DNA damage. Following the induction of DNA damage, Tip60 has also been shown to associate with ATM. In addition, Tip60 is an indispensable regulator of p53 function and acetylates transcription factors, which may play a role in DNA damage response pathways. Tat may influence all of these events, because it inhibits Tip60 activity and regulates Tip60 degradation.

The cellular DNA damage response to DSBs is controlled by three related serine/threonine kinases: ATM (ataxia telangiectasia mutated), ATR (ATM and Rad3 related) and DNA-PK (DNA-dependent protein kinase). These key players of the DNA damage response are recruited to DSB sites by other proteins, some of which appear to serve as sensors of DSBs (Figure 1). The mechanism of recruitment is specific to each kinase. ATM is recruited by the Mre11/Rad50/Nbs1 (MRN) complex [28,29] by binding to the carboxy-terminal motif of Nbs1 (product of the Nijmegen breakage syndrome gene) and in turn phosphorylates the Nbs1 protein. The Mre11 component of the complex exhibits nuclease activity necessary for DSB repair (see below, [30-34]). Current models propose that ATM-dependent Nbs1 phosphorylation controls this process [35]. The recruitment of ATR to the sites of DNA damage depends on both ATRIP (ATR-interacting protein) and replication protein A (RPA, [28,36,37]). RPA-coated single-strand DNA (ssDNA) stimulates ATR binding to the carboxy-terminal motif of ATRIP. Nevertheless, despite binding to ssDNA, ATR is also involved in DSB repair. It has been shown recently that ATR is recruited to sites of DSBs in ATM- and MRN-dependent manner [35,38]. It appears that the MRN complex processes DSBs to create the RPA-coated ssDNA, which is needed for ATR recruitment. However, the ATR-mediated DSB response is apparently restricted to the S and G2 cell cycle phases [35]. DSBs not only lead to recruitment of ATM and ATR to the sites of DNA damage, but also to their activation [39]. Activated ATM phosphorylates the Chk2 kinase and p53. Phosphorylation of these proteins leads to either p53-dependent (in G1) or p53-independent growth arrest (in S and G2 cell cycle phases). ATR, on the other hand, phosphorylates the Chk1 protein, which, together with Chk2, again induces cell cycle arrest, allowing time for DSB repair [40]. Finally, the third member of the group, DNA-PK, is a trimer consisting of the ATM- and ATR-related DNA-dependent protein kinase catalytic subunit (DNA-PK_CS_), and the Ku80/Ku70 heterodimer [41]. DNA-PK_CS _is recruited to the sites of DSBs by binding to the carboxy-terminal motif of the Ku80 (also referred to as Ku86) protein, subsequently, DNA-PK is activated [42]. Unlike ATM and ATR however, DNA-PK does not appear to be required for cell cycle arrest, and it's action is restricted to the site of DSB [41].

DSB repair is mediated by two major repair systems in mammalian cells. Similar to yeast, mammalian cells can perform homologous recombination (HR) [43]. In this process, a broken DNA molecule can be repaired by copying from an unbroken sister chromatid template, which is most effective following S-phase. The second major pathway, called non-homologous end joining (NHEJ), only a minor pathway in yeast, but the most significant pathway of DSB repair in mammalian cells [44]. NHEJ joins DSBs without the requirement for homology, by bringing together blunt ends of the broken double helix. However as a result of this mechanism, NHEJ is more error prone than HR.

In addition to their role in the activation of cell cycle checkpoints, the ATM and ATR kinases are directly involved in DSB repair. They phosphorylate the BRCA1 protein and the histone H2A variant termed H2AX [45-51]. BRCA1 is an essential player in homologous double-strand break DNA repair and physically associates with proteins implicated in this DNA repair system, including Rad51 [50].

The phosphorylated histone H2AX, termed γH2AX, appears very early at the sites of DNA damage in chromatin [52,53]. It is believed that this histone modification is required for the accumulation of DNA repair proteins, such as Rad51, at the sites of DNA damage [54]. Phosphorylation occurs in the C-terminal tail of this histone, and also a consensus site for PI-3K-related kinases. ATM is a principal kinase responsible for H2AX phosphorylation at the sites of DSBs, but ATR, and also DNA-PK, have been suggested to play a role in γH2AX formation as well [46,51]. Finally, as noted above ATM phosphorylates Nbs1 [49,55-57]. This phosphorylation is required for DNA damage response, possibly by enhancing homologous recombination [35,55-58]. Similar to ATM, it has been shown that ATR may phosphorylate Nbs1 [59].

In contrast to ATM and ATR, DNA-PK is a critical component of the NHEJ pathway. NHEJ is thought to be effective at any time during the cell cycle [60,61], but, unlike HR, may be most efficient during G1/early S phase when a peak in DNA-PK activity is observed [62]. Other core NHEJ components include the Artemis protein, and DNA ligase IV in association with Xrcc4 [41]. The Xrcc4/Ligase IV complex functions as a tetramer and is required for the final ligation of DNA ends [63,64]. The manner in which these, and other proteins that participate in NHEJ assemble at the sites of DNA lesions, how their activities are coordinated, and details of their catalytic mechanisms have yet to be fully understood. However, it has been shown that DNA-PK may phosphorylate linker histones and this process may lead to localized histone H1 release, coupled with the recruitment of Xrcc4/Ligase IV complex [41,65]. The Artemis protein is an ATM substrate and thus seems to link the ATM pathway and NHEJ [66,67]. The ATM-dependent phosphorylation increases the Artemis exonuclease activity and thus may help processing of DNA ends during the DSB repair [67].

## Double-strand break DNA repair and cancer

It has been known for a long time that patients carrying mutations in certain DNA repair genes also suffer from an increased incidence of cancer [68]. One example of such mutation is ataxia telangiectasia (A-T), which results from the ATM gene mutations, as noted above. A-T patients suffer from a range of tumors [69]. Likewise, a subset of breast cancer patients carries mutations in an ATM substrate, the BRCA1 protein [70]. In addition, it has been shown recently that a deletion of the H2AX gene in mice results in an increased incidence of tumors in p53-deficient background [71]. These findings place the ATM kinase and at least some of its substrates into a class of caretaker genes, or guardians of the genome, which offer protection from cancer-causing mutations.

Only a small fraction of population carries ATM or BRCA1 mutations [69,70]. Thus, it has not been clear what role these genes play in the development of cancer in patients that do not carry a mutation in these genes. However, two very recent publications in *Nature *showed that clinical specimens from different stages of human tumors of the urinary tract, lung, colon and breast express activated markers of the ATM- and ATR-dependent DNA damage response [72,73]. These markers are phosphorylated substrates of ATM and ATR proteins. In addition, it has been shown that expression of several oncogenes induces ATM- and ATR-dependent DNA damage response in cultured cells. These and other data indicate that early in tumorigenesis, before malignant conversion, human cells activate the ATM- and ATR-dependent DNA damage response, which delays or prevents cancer [72]. Mutations that inactivate this response, may allow cell proliferation, increased genomic instability and tumor progression. The ATM and ATR pathway thus serves as a crucial cellular anti-cancer barrier.

In contrast to ataxia telangiectasia, no such cancer-prone disease was associated with NHEJ. One likely reason is that a mutation in NHEJ has such debilitating effects that most carriers of this mutation die before they reach the stage when cancer could develop. However, NHEJ genes have been the subject of animal studies and transgenic knock out models were developed, Ku80 knock out and Xrcc4 knock out being examples discussed here.

Mice lacking Ku80 are viable, but develop severe immunodeficiency, which seems to be the most pressing problem in these animals [74]. Xrcc4-deficient mice also show a severe deficit in lymphoid development, however, they die in late embryonic development due to widespread neuronal apoptosis. Interestingly, a lack of p53 can "rescue" Xrcc4-deficient mice, but this is a short reprieve, since these double-deficient mice develop B-cell lymphomas at an early age, leading to their death within three months after birth [75]. A similar phenotype was observed for Ku80 -and p53-deficiecint mice [76]. Cells from these animals exhibit severe genomic instability, resulting in chromosomal rearrangements [76]. Thus, similar to ATM and ATR, NHEJ proteins serve as guardians of the genome, protecting genomic stability.

Taken together, ATM-, ATR-, and NHEJ-dependent DNA damage response plays a central role in tumorigenesis and agents that disrupt this response may lead to the development of cancer.

## Tat protein and the human cell

Tat is a cationic 86–101 amino acid polypeptide encoded by HIV-1 [77]. Tat functions as a transacting transcriptional activator and is involved in initiation of transcription and RNA elongation involving cellular proteins and the TAR region of HIV-1 RNA [10,78,79]. It was noted early on that Tat is released from HIV-1-infected cells [59] and can be detected in the serum of HIV-1-infected individuals at concentrations ranging from 0.1–1 ng/ml [80]. The extracellular Tat protein binds to cell surfaces. This binding is mediated by electrostatic forces, and also by binding to integrin and chemokine receptors [81,82]. In addition, Tat can be taken up by human cells and localizes to the nucleus [9,83]. The Tat protein induces different biological responses in diverse target cells. For example, Tat is known to induce expression of chemokine receptors on T-cells and is also known to act as a neurotoxin. Some of these effects are mediated by binding to cell surface receptors, whereas others could be possibly mediated by its interactions with cellular proteins within the cell nucleus [14].

## Tat and DNA repair

Although interactions of Tat with cellular proteins are widely studied, little is known about effects of Tat on cellular DNA repair. Tat was shown to induce the expression of the DNA polymerase beta gene, which encodes a key protein in the DNA base-excision repair pathway [84]. Expression of this gene was shown to be increased in AIDS-related lymphomas [84].

In addition to regulation of DNA polymerase beta gene, Tat was also implied to play a role in DSB DNA repair, since cellular extracts containing the Tat protein have a reduced ability to re-join linearized DNA [85]. This finding implies a role for Tat in the regulation of the DNA damage response to double-strand DNA breaks.

### Tat and DNA-PK

An additional piece of evidence for the Tat role in the suppression of double-strand DNA break repair came from Tat-expressing rhabdomyosarcoma cell lines [86]. In these cells, Tat induced increased sensitivity to ionizing radiation, which is a major source of double-strand DNA breaks. In addition, a comet assay and an analysis of γH2AX foci revealed reduced repair of double-strand DNA breaks. Lastly, Tat-expressing cells showed an extended G2/M growth arrest in response to ionizing radiation, again indicating a defect in DSB repair. A microarray analysis showed that Tat repressed expression of DNA-PKcs, which is a major NHEJ component, as noted above [86]. Taken together, these data show that Tat blocks cellular DSB repair, and this may be associated with low endogenous levels of DNA-PKcs. However, Tat may affect DSB repair in yet another way, as described below.

### Tat and Tip60

The cellular protein Tip60 (Tat interactive protein) was originally discovered as a protein interacting with HIV-1 Tat [87]. However, since that time, Tip60 became a major object of study in its own right, since it influences critical cellular processes even in the absence of Tat [88-94]. Moreover, Tip60 is linked to a number of cancers [95]. Tip60 possesses histone acetyltrasferase (HAT) activity and appears to target lysine residues of nucleosomal H2A and H4 [96]. Tip60 is predominantly nuclear and has both transcriptional and DNA repair functions, which will be described in detail below.

Tip60 is involved in multiple steps of the cellular DNA damage response. Similarly to p53, Tip60 is a target of the human Mdm2 protein, which catalyzes Tip60 ubiquitination and proteosomal degradation [91]. Second, like p53, Tip60 also accumulates after DNA damage [92]. However, the strongest piece of evidence for Tip60 interaction with the p53 pathway came from a large RNAi screen, which showed that Tip60 was essential to p53-dependent G1/S growth arrest [88]. Tip60 is also involved in up-regulation of p53-responsive genes for p21 and GADD45 proteins and Mdm2 [97]. As shown in Figure 1, p53 induces either growth arrest or apoptosis. The molecular mechanism underlying this p53 choice is not yet fully elucidated. However, it has been shown very recently that Tip60 acetylates the lysine 120 residue of p53 [94]. This modification is required for p53-induced apoptosis, but dispensable for p53-mediated growth arrest [94]. Taken together, Tip60 is an indispensable regulator of p53 function.

The second point of interaction of Tip60 with DSB repair is the ATM kinase (Figure 1). ATM autophosphorylation is required for separation of the inactive ATM dimmer into active monomers [39]. It has been reported recently that Tip60 forms a stable complex with ATM and in response to DSBs acetylates the ATM protein [93]. Acetylation by Tip60 is required for activation of the ATM kinase, autophosphorylation and phosphorylation of downstream ATM substrates, such as p53 and Chk2 [93]. The interaction of Tip60 and ATM is not regulated by DNA damage and activation of Tip60 by DNA damage and the recruitment of the ATM-Tip60 complex to sites of DNA damage is independent of ATM's kinase activity [93]. Tip60 is thus involved in regulation of and acts upstream of a critical component of cellular DSB repair. However, the ATM-Tip60 interaction remains to be fully understood, since it has been reported that Tip60 knockdown in Drosophila does not impair phosphorylation of H2Av (equivalent of human H2AX, which is a major ATM substrate) [98]. Finally, it has been shown very recently that transgenic mice that are heterozygous for a Tip60 knockout allele (*Tip60*^+/-^) exhibit severely impaired oncogene-induced DNA damage response, which is ATM- and ATR-dependent [[95] see above also]. Surprisingly, cells from *Tip60*^+/- ^did not have a general deficit in DNA damage response to DSBs (ionizing radiation), which could be inferred based on the defect in oncogene-induced DNA damage response. These latter data thus seemingly contradict the above report indicating that Tip60 is required for ATM activation in response to DSBs. However, it has to be pointed out that the *Tip60*^+/- ^mice still had one active Tip60 allele and it is thus possible that what is observed is a dose-dependent effect and an additional Tip60 inhibition is required to see its role in DNA damage response to DSBs. Unfortunately, homozygous embryos that lack Tip60 die before implantation, thus complicating the analysis. In summary, two lines of evidence suggest that Tip60 interacts with ATM and regulates the ATM-dependent DNA damage response, although some aspects of this interaction remain to be clarified.

Cellular DNA is packaged together with histones into chromatin, and the chromatin structure may affect sensing and repair of DSBs. Acetylation of histones is thought to "open" the chromatin structure DNA to DNA repair proteins. Tip60 was shown to acetylate histone H4 at the DSB sites [92]. Lack of Tip60-dependent H4 acetylation can be apparently complemented by chromatin relaxation by different means, indicating that the Tip60 increases chromatin accessibility. Acetylation of histone H4 is thus yet another point of interaction of Tip60 with DSB repair. Finally, Kusch *et al*. reported that Tip60 is required in Drosophila for selective histone exchange at DSB sites [98]. Specifically, Tip60 acetylates phosphorylated histone H2Av (as noted above, H2AX equivalent) and exchanges it with unmodified H2Av. Tip60 thus participates in DSB repair by yet another mechanism, although the role of acetylation and subsequent exchange of H2Av in DSB repair has yet to be fully understood.

### Tat and Tip60 interaction and consequences

As noted above, Tip60 was originally identified as a Tat-intereacting protein [87]. Tip60 does not affect Tat-dependent transactivation of the HIV-1 LTR promoter [99]. However, Tat blocks the HAT activity of Tip60 [99]. Interestingly, a new mechanism of Tip60 neutralization by Tat has been recently reported, where Tat induces polyubiquitination and degradation of Tip60 [89]. Tip60 ubiquitination is dependent in this process on the newly reported p300/CBP-associated E4-type ubiquitin-ligase activity [89]. Therefore, the presence of the Tat protein is likely to inhibit Tip60 function (Figure 1). Given the multiple roles of Tip60 in DSB repair, it seems likely that Tat may exert effects on DSB repair. These effects may be detrimental to DSB repair and eventually lead either to apoptosis or to mutations when cells are exposed to DSB-inducing agents or environmental factors.

## Summary

In this article, we summarize several functions of Tat and its interacting proteins in cellular DNA repair. We suggest that Tat may, by regulating its cellular targets, affect cellular DSB repair, the consequences of which could be genomic instability, which may give rise to mutations and contribute to development of cancer. Experiments are underway in our laboratories to investigate Tat role(s) in DSB repair. It is hoped that this review will stimulate further study in this underexplored, yet potentially important field.

## Authors' contributions

GN and RD conceived of the idea and co-wrote the manuscript. JAS provided helpful comments, created the model for Tip60 role in DSB repair and participated in the preparation of the manuscript.
